# Supplementary material for: A New Variant of the aadE-sat4-aphA-3 Gene Cluster Found in a Conjugative Plasmid from a MDR Campylobacter jejuni Isolate
Source: Antibiotics (Basel). 2022 Mar 30;11(4):466. doi: 10.3390/antibiotics11040466 (PMC9032879; doi:10.3390/antibiotics11040466)
Supplement: Supplementary file 1 [file antibiotics-11-00466-s001.zip › Figure S1.pdf]

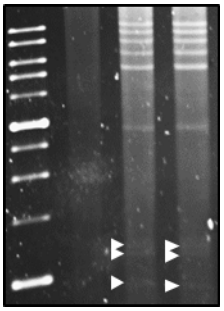

Figure S1: Overexposed caption from the gel shown in Fig. 2A, to improve detection of the three lowest mass bands indicated with white arrowheads.
