# Supplementary material for: A New Variant of the aadE-sat4-aphA-3 Gene Cluster Found in a Conjugative Plasmid from a MDR Campylobacter jejuni Isolate
Source: Antibiotics (Basel). 2022 Mar 30;11(4):466. doi: 10.3390/antibiotics11040466 (PMC9032879; doi:10.3390/antibiotics11040466)
Supplement: Supplementary file 1 [file antibiotics-11-00466-s001.zip › Table S1.pdf]

Table S1. Primers used in this study

**Target gene**	**Primer**	**Sequence**	**Reference**
tet(O)	tet(O)F	GCGTTTTGTTTATGTGCG	Obeng et al., 2012 [45]
	tet(O)R	ATGGACAACCCGACAGAAG	
aph(3')-IIIa	CJ-IIIF	GAAAGCTGCCTGTTCCAAAG	Crespo et al., 2016 [46]
	CJ-IIIR	ATGTTGCTGTCTCCCAGGTC	
wlaN	wlanF	TGCTGGGTATACAAAGGTTGTG	Koolman et a., 2015 [47]
	wlanR	AGGTCCATTACCGCATACCA	
